# IL-13-induced proliferation of airway epithelial cells: mediation by intracellular growth factor mobilization and ADAM17

**DOI:** 10.1186/1465-9921-8-51

**Published:** 2007-07-09

**Authors:** Brian W Booth, Tracy Sandifer, Erika L Martin, Linda D Martin

**Affiliations:** 1 Department of Molecular Biomedical Sciences, North Carolina State University, Raleigh, NC, USA; 2 Mammary Biology and Tumorigenesis Laboratory, National Cancer Institute, National Institutes of Health, Bethesda, MD, USA; 3 Department of Epidemiology, School of Public Health and Community Medicine, University of Washington, Seattle, WA, USA

## Abstract

**Background:**

The pleiotrophic cytokine interleukin (IL)-13 features prominently in allergic and inflammatory diseases. In allergic asthma, IL-13 is well established as an inducer of airway inflammation and tissue remodeling. We demonstrated previously that IL-13 induces release of transforming growth factor-α (TGFα) from human bronchial epithelial cells, with proliferation of these cells mediated by the autocrine/paracrine action of this growth factor. TGFα exists as an integral membrane protein and requires proteolytic processing to its mature form, with a disintegrin and metalloproteinase (ADAM)17 responsible for this processing in a variety of tissues.

**Methods:**

In this study, normal human bronchial epithelial (NHBE) cells grown in air/liquid interface (ALI) culture were used to examine the mechanisms whereby IL-13 induces release of TGFα and cellular proliferation. Inhibitors and antisense RNA were used to examine the role of ADAM17 in these processes, while IL-13-induced changes in the intracellular expression of TGFα and ADAM17 were visualized by confocal microscopy.

**Results:**

IL-13 was found to induce proliferation of NHBE cells, and release of TGFα, in an ADAM17-dependent manner; however, this IL-13-induced proliferation did not appear to result solely from ADAM17 activation. Rather, IL-13 induced a change in the location of TGFα expression from intracellular to apical regions of the NHBE cells. The apical region was also found to be a site of significant ADAM17 expression, even prior to IL-13 stimulation.

**Conclusion:**

Results from this study indicate that ADAM17 mediates IL-13-induced proliferation and TGFα shedding in NHBE cells. Furthermore, they provide the first example wherein a cytokine (IL-13) induces a change in the intracellular expression pattern of a growth factor, apparently inducing redistribution of intracellular stores of TGFα to the apical region of NHBE cells where expression of ADAM17 is prominent. Thus, IL-13-induced, ADAM17-mediated release of TGFα, and subsequent epithelial cell proliferation, could contribute to the epithelial hypertrophy, as well as other features, associated with airway remodeling in allergic asthma.

## Background

Growth factors and cytokines serve integral functions in physiological processes as diverse as proliferation, differentiation, angiogenesis, immune responses and disease progression [1-3]. In a process impacting many cell types such as an immune response, the relationship between cytokines and growth factors can influence the response of tissues that become surrounded by an inflammatory milieu [3]. Similarly, cytokines and growth factors serve to ultimately enhance or resolve inflammation-induced changes in biological structures [4,5]. Such a coordinated relationship between the cytokine interleukin-13 (IL-13) and the growth factor, transforming growth factor-α (TGFα), was demonstrated previously by our laboratory in normal human bronchial epithelial (NHBE) cells. In these cells, IL-13 was found to induce proliferation via the autocrine/paracrine activity of epithelium-derived TGFα [6].

IL-13, produced by CD4^+ ^T cells, is categorized as a Th2 cytokine based on its roles in immune function [7]. IL-13 is also known to be a central mediator of the allergic asthmatic phenotype, exerting numerous effects on airway epithelial cells [8]. Specifically, IL-13 has been shown to play a role in the development of mucous cell hyperplasia [9-11], in activating matrix metalloproteinases [12], and in inducing expression of epithelium-derived growth factors (i.e. TGFα [6], TGFβ [13]) and chemokines (i.e. eotaxin [14], MCP-3 [15]). These released factors, in turn, affect neighboring epithelial cells as well as other cell types within the airway walls such as fibroblasts and smooth muscle cells [16]. While it is well documented that epithelial cells, including those of the airways, produce and release growth factors [17], the mechanism, or mechanisms, regulating cytokine-induced release of growth factors has not been fully elucidated.

TGFα is a growth factor that helps control essential biological processes such as development, differentiation, and proliferation [18-20], with its overexpression contributing to a variety of disease states. Specifically, overexpression of TGFα has been implicated in the development of mammary, squamous, and renal carcinomas, melanomas, hepatomas, glioblastomas [21,22], and in the induction of pulmonary fibrosis or emphysema [23,24].

The release of mature TGFα requires proteolytic cleavage of a membrane-associated pro-peptide. This process, termed shedding, is usually accomplished by the ADAM (adisintegrin and metalloproteinase) family member, TNFα converting enzyme (TACE or ADAM17) [25]. ADAM17 appears to be activated by protein kinase C (PKC) [26], nitric oxide (NO) [27] and extracellular signal-regulated kinase (Erk) [28]. Although cytokines are known to activate PKC, NO and Erk in a variety of cells [29], direct cytokine-induced activation of ADAM17 has yet to be documented. ADAM17 does, however, have the capacity to mediate cytokine-inducible events such as MUC5AC expression, as demonstrated in an airway epithelial cell line (NCI-H292) [30]. Furthermore, IL-13-induced mucin gene and protein expression can be blocked by a broad-spectrum inhibitor of MMP/ADAM in differentiated NHBE cells [31].

ADAM17 is known to be expressed on the surface of cells, and has been observed in perinuclear compartments as is the ADAM17-cleavable protein, TNFα [32]. Another ADAM17 target, TGFα, also has been found stored in intracellular granules in monocytes, neutrophils [33], and eosinophils [34]. It is not known, though, whether these intracellular stores of growth factor are mobilized in response to stimuli that induce shedding.

In this study, we use primary NHBE cells differentiating in air/liquid interface (ALI) culture to explore potential relationships between IL-13, ADAM17, and TGFα in the mechanism controlling IL-13-induced proliferation. Specifically, we demonstrate that IL-13-induced proliferation of NHBE cells requires ADAM17; however, the mechanistic link between IL-13 and TGFα shedding seems to involve more than a simple increase in ADAM17 activity. Rather, we show that IL-13 appears to mobilize intracellular TGFα to the apical region of the cells where the cleavage enzyme ADAM17 is expressed in abundance.

## Materials and methods

### Cell culture and experimental protocol

NHBE cells (Cambrex, Walkersville, MD) were grown on Transwell membranes as described previously [35]. Media was changed every other day until the cells reached confluence, at which time the apical medium was removed to establish an ALI. Thereafter, the basolateral medium was changed daily. All experimentation was carried out on day 7–9 after ALI establishment. At this point, mature secretory cells are present in these differentiating cultures and the cells respond with maximal proliferation to IL-13 (10 ng/ml) as determined previously [6]. Concentrations of TGFα (5 ng/ml) and neutralizing antibodies (0.2 μg/ml) used were based on studies utilizing similar compounds in NHBE cells ([6]; X Fu and LD Martin, unpublished results). A range of concentrations of rhADAM17 (50 - 0.1 ng/ml) as well as TIMP1 and TIMP3 (100 - 0.5 μg/ml; R&D Systems, Minneapolis, MN) were examined for effectiveness in modulating IL-13-induced proliferation or TGFα shedding in NHBE cells. The lowest possible concentrations that yielded repeatable results with little impact on constitutive growth or growth factor release were used for final experiments in this study. All experiments were repeated a minimum of three times using cells from at least two human donors (except the RT-PCR which was done once). One representative experiment is shown in each Figure.

### ELISA

Following experimental treatments, media samples were collected and analyzed with commercially-available TGFα or IL-8 ELISA kits according to manufacturer's instructions (R&D Systems, Minneapolis, MN).

### Proliferation assays

[^3^H]-thymidine incorporation assays were performed as described previously [6]. Cells were exposed for 24 hrs to IL-13 (10 ng/ml) and/or specific reagents as described. To perform manual cell counts, NHBE cells were liberated from the Transwell membranes with warm Versene (Invitrogen, Grand Island, NY) for 5–10 min at 37°C and counted using a hemacytometer.

### Antisense assays

Antisense oligonucleotides were utilized following a protocol modified from Li et al [36]. Briefly, NHBE cells in ALI culture were exposed to varying concentrations of antisense oligonucleotides directed against ADAM17, scrambled oligonucleotides as a control, or transfection reagent alone (FuGene6; Roche, Indianapolis, IN). All cells were treated for 3 days with the oligonucleotides, with FuGene6 added only on the first day at the manufacturer's suggested concentration. On the third day, the cells were exposed to IL-13, media (control) or TGFα for 24 hrs, with media samples collected and cells counted. Phosphorothioate-modified oligonucleotides were synthesized by Invitrogen (Rockville, MD). ADAM17 antisense sequence was 5'-CCG CCT CAT GTT CCC GT-3' [Genbank: NM_003183]. The scrambled sequence was 5'-TGC GCC ATC TCG CTC TC-3'.

### Immunoprecipitation

Total protein was extracted from NHBE cells using RIPA buffer containing Roche Complete protease inhibitor cocktail (1 mM EDTA; 1% NP-40; 0.5% sodium deoxycholate, 0.1% SDS, 30 μg/ml pancreas extract, 3 μg/ml pronase, 0.8 μg/ml thermolysin, 1.5 μg/ml chymotrypsin, 0.2 μg/ml trypsin, and 1.0 mg/ml papain). These lysates were incubated overnight with primary antibody at 4°C with shaking. A 50% slurry of Protein A was then added and incubated for 3 hrs. The resulting pellet was washed 5 times in buffer and mixed 1:1 with 2× SDS gel loading buffer (100 mM Tris-Cl, pH 6.8; 4% SDS, 0.2% bromophenol blue, 20% glycerol, 200 mM β-mercaptoethanol). Western analysis was then performed.

### Western analysis

Total protein in 2× SDS gel loading buffer was boiled for 5 min, and separated via SDS-PAGE on 10–20% precast gradient gels (Bio-Rad, Hercules, CA). Proteins were transferred to a nitrocellulose membrane (Bio-Rad, Hercules, CA) that was then blocked in 5% nonfat milk/PBS for 1 hr at room temperature. Membranes were hybridized with primary anti-ADAM17 antibody (R&D Systems, Minneapolis, MN) at a concentration of 1:1000 in 5% nonfat milk/PBS overnight at 4°C. The membranes were then washed twice (30 min each) with 0.01% Tween-20/PBS at room temperature. After the second wash, the membrane was exposed to HRP-conjugated secondary antibody diluted 1:5000 in 5% nonfat milk/PBS for 45 min at room temperature. Washes were repeated and bands visualized with ECL (Amersham, Buckinghamshire, UK). The blots were stripped using a commercially available kit (Chemicon International, Temecula, CA) and then rehybridized with an anti-actin primary antibody (Santa Cruz Biotechnology, Santa Cruz, CA) to verify equal protein loading.

### RT-PCR

Total RNA was extracted from NHBE cells with TRI Reagent (Sigma, St. Louis, MO) and reverse transcribed using specific oligonucleotides and the First Strand cDNA Synthesis Kit for RT-PCR (AMV) (Roche, Indianapolis, IN) in accordance with manufacturer's guidelines. Effort was made to use the amount of cDNA in each PCR that would provide a product in the linear range of the reaction in 35 cycles. PCR reactions were carried out using Taq polymerase (Boeringher Mannheim, Mannheim, Germany) in a Perkin Elmer GenAmp PCR System 2400. PCR products were separated by electrophoresis through a 2% agarose gel and visualized by staining with ethidium bromide. Primers used were ADAM17 forward-ACCTGAAGAGCTTGTTCATCGAG, ADAM17 reverse-CCATGAAGTGTTCCGATAGATGTC [Genbank: NM_003183]; β-actin forward-TCGACAACGGCTCCGGCA, β-actin reverse-CGTACATGGCTGGGGTGT [Genbank: BC014861].

### Confocal microscopy

At each time point, 2 control cultures were exposed to media and 2 experimental cultures to IL-13 (10 ng/ml). Following treatment, the NHBE cells were fixed on the Transwell inserts with 4% formalin. All staining was carried out in the Transwell inserts. Cells were washed with PBS, permeabilized with 0.2% Triton X-100 in PBS, and reacted with primary antibodies, either anti-TGFα or anti-ADAM17, followed by a 45 min incubation in the dark with appropriate secondary antibodies tagged with Alexa 488 (for use with anti-ADAM17) or Alexa 594 (for use with anti-TGFα) (Molecular Probes, Eugene, OR). Membranes containing the cells were then removed from the Transwell inserts and mounted on glass slides in Vectashield mounting media (Vector Laboratories, Burlingame, CA). Cells were visualized with a Nikon Eclipse TE2000-E confocal microscope via a Plan Apo 60× water immersion objective. The entire experiment, from cell growth through microscopy, was repeated 3 times, resulting in 6 samples per experimental and 6 samples per control, time point. Each sample was divided into quadrants and 250 to 300 cells per quadrant were examined qualitatively to gain a general understanding of the expression patterns at each time point.

### Confocal quantitative analyses

Six to nine scans per control or experimental time point were chosen randomly from the captured Z-stack confocal microscopy images. Five to 10 cells per scan were examined. Three areas [apical/middle/basal] within each cell were inspected to determine whether more TGFα or more ADAM17 was expressed in each area. The Z-stack images had been generated using a constant Z-stack interval. In each Z-stack, the first image was from just above the transwell membrane at the basal cellular surface and the last image was at the cell's apical surface. Thus, "apical" and "basal" refer to the apical-most and basal-most images in the Z-stack from a single cell, while "middle" is defined as the image halfway between the apical-most and basal-most images from a single cell. With examination of approximately 100 cells (50 control and 50 experimental) per time point, about 97% of the cells were found to have essentially two expression patterns [apical/middle/basal]: [TGFα/TGFα/ADAM17] or [ADAM17/ADAM17/TGFα ]. Using only these 97% of cells, final percentages of cells exhibiting each pattern were calculated.

### Statistical analysis

Experimental data were analyzed for significance by one-way analysis of variance (ANOVA), with post-test correction for multiple comparisons where appropriate. Differences between treatments were considered significant at p < 0.05. Data are shown as mean ± standard error of the mean (SEM).

## Results

### ADAM17 induces TGFα-mediated proliferation of NHBE cells

**Figure 1:**
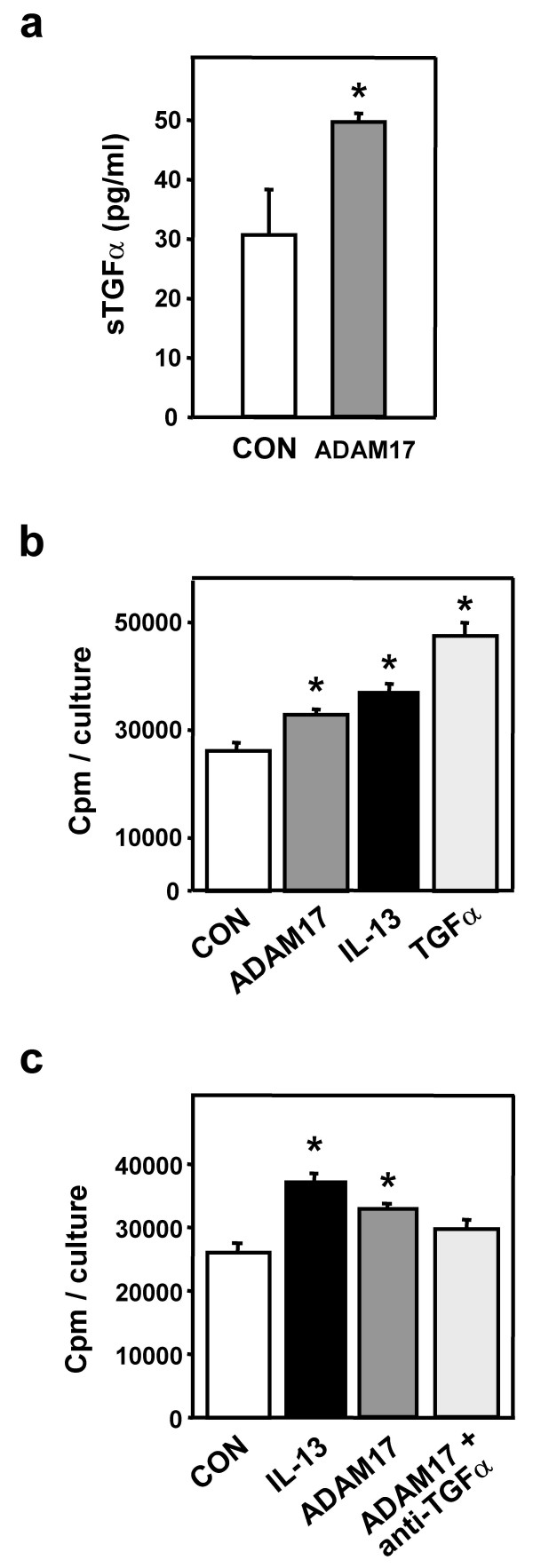
**ADAM17-induced proliferation is mediated by TGFα**. **a) **NHBE cells were treated with rhADAM17 (10 ng/ml) for 1 hr after which surrounding medium was analyzed for the presence of TGFα by ELISA (n = 3, *p < 0.05 vs. CON). **b) **NHBE cells were treated with rhADAM17 (10 ng/ml), IL-13 (10 ng/ml), or TGFα (5 ng/ml) for 24 hrs. [^3^H]-thymidine incorporation was used as a measure of proliferation (n = 6, *p < 0.05 vs. CON). **c) **NHBE cells were treated with IL-13 (10 ng/ml), ADAM17 (10 ng/ml) or ADAM17 plus neutralizing anti-TGFα antibody (0.2 μg/ml) for 24 hrs, with [^3^H]-thymidine incorporation used as a measure of proliferation (n = 6, *p < 0.05 vs. CON).

Research from our laboratory indicates that IL-13 initiates proliferation of NHBE cells via a TGFα/EGFR (epidermal growth factor receptor) autocrine/paracrine growth loop [6]. Since ADAM17 is known to cleave membrane-inserted pro-TGFα to its mature form in a number of cell types [25,37,38], we determined whether ADAM17 could act similarly in NHBE cells to mediate proliferation in a TGFα-dependent manner. Treatment of NHBE cells with exogenous recombinant human (rh) ADAM17 resulted in an increase of soluble TGFα in the surrounding medium (Fig. 1a). ADAM17 also induced cellular proliferation as did IL-13 and TGFα (Fig. 1b). These results indicate that NHBE cells express TGFα on the extracellular membrane in a form that is amenable to proteolytic cleavage by ADAM17. Next we determined if the proliferation observed following exposure to rhADAM17 was occurring via cleavage of surface expressed TGFα, rather than via cleavage of another growth factor. The addition of neutralizing anti-TGFα antibody attenuated the proliferative effect induced by exogenous rhADAM17 (Fig. 1c) suggesting that rhADAM17 cleaves surface-expressed TGFα, that in turn induces proliferation of the epithelial cells.

### ADAM17 mediates IL-13-induced proliferation of NHBE cells

**Figure 2:**
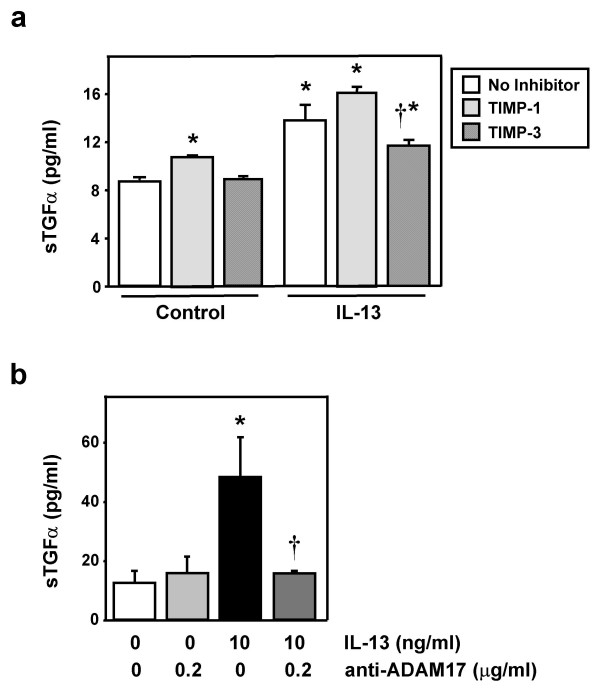
**Inhibitors of ADAM17 attenuate IL-13-induced shedding of TGFα**. NHBE cells were exposed to control media, inhibitors of ADAM17, IL-13 or IL-13 plus inhibitors for 1 hr. **a) **NHBE cells were exposed to either control media (no inhibitor), TIMP-1 or TIMP-3 (both at 2 μg/ml) for 30 min prior to treatment with IL-13 (10 ng/ml) or control media. The inhibitors were also included during the treatment period. After the 1 hr treatment, supernatants were examined for TGFα shedding via ELISA (n = 4, *p < 0.05 vs. corresponding control, †p < 0.05 vs. IL-13 alone). Light gray bars = TIMP-1; Dark gray bars = TIMP-3. **b) **NHBE cells were exposed to control media, anti-ADAM17 antibodies, IL-13, or IL-13 plus anti-ADAM17 for 1 hr. Supernatants were then examined for shed TGFα via ELISA (n = 6, *p < 0.05 vs. media control, †p < 0.05 vs. IL-13 alone).

After determining that exogenous ADAM17 can induce cellular proliferation mediated by TGFα in NHBE cells, we determined whether endogenous ADAM17 is involved in IL-13-induced proliferation of these cells. First, the effects of various inhibitors of ADAM17 on IL-13-induced shedding of TGFα were examined. Tissue inhibitor of metalloproteinase (TIMP)-3 is a documented inhibitor of ADAM17 [39,40], while a related family member, TIMP-1, has been found to have no effect on ADAM17 [41]. Furthermore, the differential inhibition of ADAM17 by the two TIMPs is useful to distinguish the action of ADAM17 from that of ADAM10, whose activity can be inhibited by both TIMP-3 and TIMP-1 [41]. In the current study, TIMP-3 was found to attenuate IL-13-induced TGFα shedding, while TIMP-1 did not have an inhibitory effect (Fig. 2a). Additionally, anti-ADAM17 antibodies also blocked IL-13-induced TGFα shedding (Fig. 2b). Thus, these data support the role of ADAM17 in mediating IL-13-induced TGFα shedding in NHBE cells.

**Figure 3:**
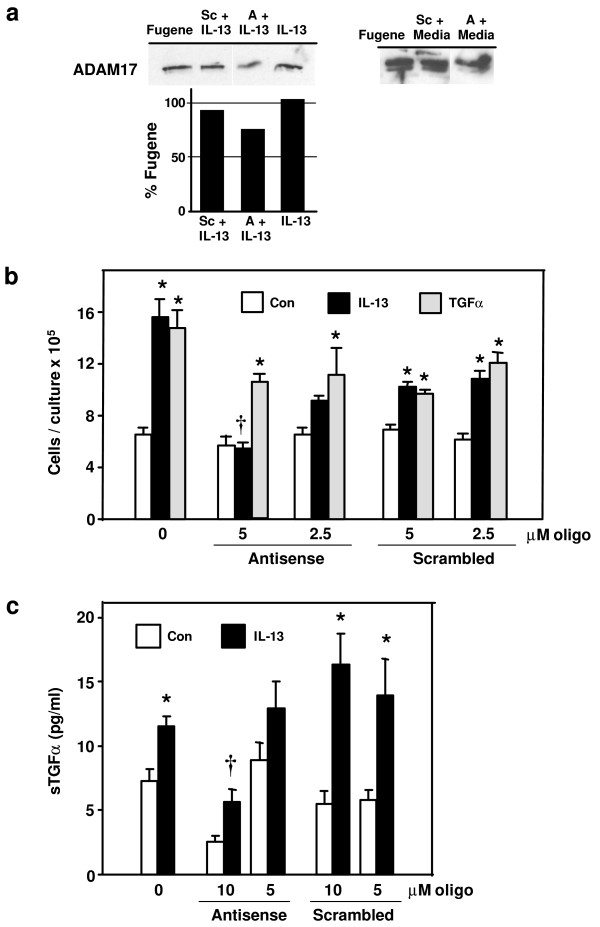
**Blocking endogenous ADAM17 inhibits IL-13-induced effects**. Antisense oligonucleotides directed against ADAM17 (antisense), or corresponding scrambled oligonucleotides (scrambled), were added to NHBE cell cultures for 2 days. Cultures containing no oligonucleotides received the transfection reagent (FuGene6) during this time. On the third day, cells were exposed to control media, IL-13 (10 ng/ml), or TGFα (5 ng/ml), with or without the addition of the scrambled or antisense oligonucleotides for 24 hrs. **a) **Total protein was extracted from a single culture from each treatment group and from the FuGene-only control group. ADAM17 was immunoprecipitated from these extracts and subjected to Western analysis (A = antisense oligonucleotides; Sc = scrambled oligonucleotides; 10 μM). The percentage of ADAM17 in experimental cultures compared to a FuGene-only exposed culture (Fugene) was determined by densitometry as indicated (left panel). The right panel was overexposed to verify the location of the two, expected ADAM17 bands. Both blots reveal decreased expression of ADAM17 in the two cultures exposed to antisense oligonucleotides. **b) **Cell number was determined as a measure of proliferation (n = 6, *p < 0.05 compared to appropriate control, †p < 0.01 compared to appropriate IL-13-treated, scrambled oligo sample), and **c) **the amount of TGFα in the supernatant was quantified via ELISA (n = 4, *p < 0.05 compared to appropriate control, †p < 0.01 compared to appropriate treated, scrambled oligo sample).

To confirm the requirement of ADAM17 in mediating IL-13-induced TGFα shedding, and to determine whether ADAM17 is similarly required for IL-13-induced NHBE cell proliferation, cells were exposed to antisense oligonucleotides directed against ADAM17 or to scrambled oligonucleotides as a control. Scrambled oligonucleotides had little effect on ADAM17 expression in a culture exposed to media and in another culture exposed to IL-13; however, in the same experiment, decreased expression of ADAM17 was easily discernible in comparable cultures exposed to antisense oligonucleotides directed against the protease (Fig. 3a). In cultures similarly exposed in this same experiment, ADAM17 antisense oligonucleotides inhibited IL-13-induced NHBE cell proliferation (Fig. 3b) and inhibited IL-13-induced, as well as constitutive, shedding of TGFα (Fig. 3c). ADAM17 antisense oligonucleotides, however, did not inhibit TGFα-induced proliferation (Fig. 3b). In all experiments, scrambled oligonucleotides had no significant effect on growth of control cells or on their constitutive release of TGFα (Figs. 3b and 3c). Furthermore, while the presence of scrambled or ADAM17 antisense oligonucleotides reduced the maximal level of proliferation inducible by IL-13 or TGFα, only the ADAM17 antisense oligonucleotides were capable of blocking IL-13-induced proliferation with specificity, as these oligonucleotides had no effect on TGFα-induced proliferation (Fig. 3b). Taken together, these results support the requirement of endogenous ADAM17 for IL-13-induced proliferation of NHBE cells, and confirm that ADAM17 plays a role in the shedding of TGFα in NHBE cells.

### IL-13-induced effects are not mediated solely via activation of ADAM17

**Figure 4:**
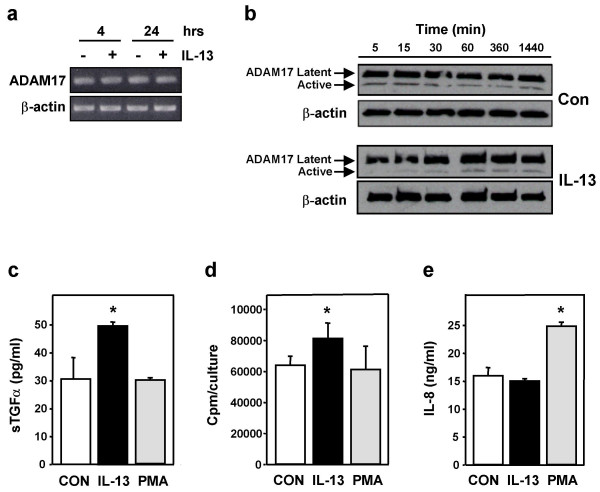
**IL-13-induced effects are not due solely to activation of ADAM17**. **a) **NHBE cells were exposed to IL-13 (10 ng/ml) or control media for 4 or 24 hrs, and steady-state mRNA levels of ADAM17 and β-actin determined via RT-PCR. Ethidium bromide-stained gels of PCR products are shown. **b) **NHBE cells were treated with control media or IL-13 for the times indicated. Total protein from these cells was examined for ADAM17 expression via Western blot. Membranes were chemically stripped and rehybridized to detect β-actin as a control for equal protein loading. **c) **NHBE cells were treated with control media, IL-13, or PMA (10 nM) for 1 hr and the supernatants examined for soluble TGFα via ELISA (n = 4, *p < 0.05 compared to control). **d) **NHBE cells were treated for 24 hrs with control media, IL-13, or PMA (10 nM), and [^3^H]-thymidine incorporation determined as a measure of proliferation (n = 6, *p < 0.05 compared to control). **e) **Secretion of IL-8 from NHBE cells was examined by ELISA following 1 hr exposure to control media, IL-13, or PMA (10 nM) (n = 6, *p < 0.05 compared to control).

Since ADAM17 appeared to mediate IL-13-induced TGFα shedding and proliferation in NHBE cells, we wanted to determine whether these effects were due to a simple IL-13-induced increase in ADAM17, or its activity. The amount of steady-state mRNA coding for ADAM17 in control or IL-13-treated cells was found to be the same following 4 or 24 hrs of treatment (Fig. 4a). Next the amount of ADAM17 protein was examined. This protein exists in two forms, an inactive, latent form and an active form [32]. Conversion to the active form requires proteolytic cleavage of the enzyme, resulting in removal of a 20-kDa section of the protein. The amount of latent ADAM17 in NHBE cells varied little in response to control media or IL-13 over a time course of 5 min to 24 hrs (Fig. 4b). The amount of active ADAM17 in control cells during this time period also varied little, while slightly less active ADAM17 was observed at early time points in IL-13-treated cells. The amount of active ADAM17 in these treated cells, however, was similar to control levels at the latter time points (1 to 24 hrs) (Fig. 4b). Thus, while IL-13 may induce a small, transient decrease in the amount of active ADAM17, the quantity of active protein is no greater than that observed in control cells at time points when IL-13 induces an increase in soluble TGFα (i.e. approximately 60 min in this study (Fig. 4c), and as early as 15 min in a previous study [6]). These data show that IL-13 does not induce a dramatic alteration in the amount of ADAM17 mRNA, latent ADAM17, or active ADAM17 in NHBE cells.

Since activation of ADAM17 and ADAM17-mediated shedding can be induced via PKC stimulation [26,42], we tried to enhance the shedding of TGFα by exposing NHBE cells to phorbol-12-myristate 13-acetate (PMA), a known activator of PKC and well-characterized inducer of TGFα ectodomain shedding [43], at a concentration shown previously to enhance TGFα shedding in a pulmonary mucoepidermoid carcinoma cell line (NCI-H292) [30]. Exposure of the NHBE cells to PMA, however, did not yield an increase in soluble TGFα (Fig. 4c) or cellular proliferation (Fig. 4d), even though IL-13 could still induce these events. The NHBE cells did respond to the PMA, however, as secretion of IL-8, a process known to be PKC-dependent in NHBE cells [44], was enhanced while IL-13 had no effect on IL-8 secretion (Fig. 4e). Thus, these results suggest that the mechanism mediating IL-13-induced release of soluble TGFα from NHBE cells differs from the PKC-mediated mechanism responsible for TGFα shedding in NCI-H292 cells, an event which appears to involve direct activation of ADAM17 by PKC [30]. Thus, it appears that although the IL-13-induced increase in TGFα shedding, as well as the IL-13-induced proliferation, is mediated by ADAM17 in NHBE cells, these events do not occur solely via an IL-13-induced increase in ADAM17 or its activity.

### IL-13 induces apical movement of intracellular TGFα

An alternate mechanism whereby IL-13 could increase the amount of TGFα shed from NHBE cells would be for the cytokine to promote the release of pre-formed, intracellular growth factor. NHBE cells are already known to release pre-formed mucin proteins (the glycoprotein component of airway mucus) upon stimulation with various inflammatory mediators [36,45]. Under such conditions, granules containing the mucin proteins are thought to be mobilized rapidly to the cell surface where the proteins are secreted [36]. To determine whether a similar mechanism mediates IL-13-induced release of TGFα, confocal microscopy was used to examine the location of TGFα and its sheddase, ADAM17, in NHBE cells exposed to IL-13 or control media over a 4-hr time course. (Quantitative results from this study are shown in Table 1.)

**Table 1:** Percentage of NHBE cells with specified patterns of TGFα/ADAM17 expression following IL-13 stimulation.

		**EXPRESSION PATTERN**	
**Time point**		**% Cells with Apical TGF**α	**% Cells with Apical ADAM17**

15 min	CON	**19**	**81**
	IL-13	**20**	**80**
30 min	CON	**18**	**82**
	IL-13	**46**	**54**
60 min	CON	**29**	**71**
	IL-13	**35**	**65**
4 hrs	CON	**12**	**88**
	IL-13	**2**	**98**

Cells were examined by confocal microscopy to determine whether expression of TGFα or ADAM17 was greater in the apical-most, middle and basal-most sections of the Z-stack images. "Expression pattern" refers to expression noted within [apical/middle/basal] regions on an NHBE cell. "Apical TGFα " refers to a pattern of [TGFα/TGFα/ADAM17]. "Apical ADAM17" refers to a pattern of [ADAM17/ADAM17/TGFα].

**Figure 5:**
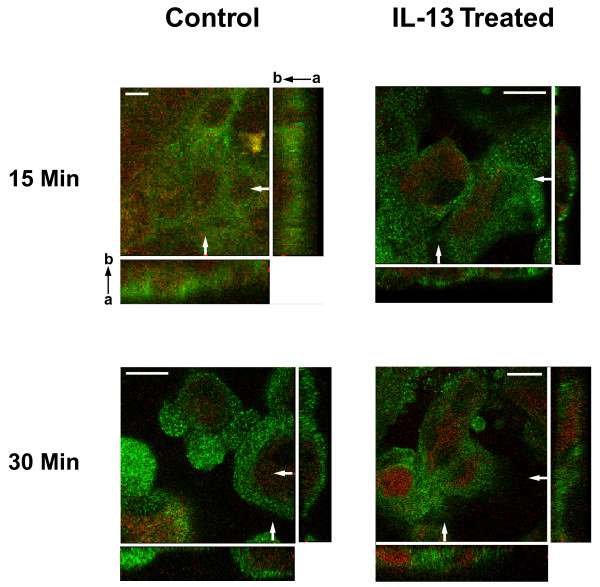
**TGFα and ADAM17 expression patterns are consistent with IL-13-induced movement of TGFα**. Confocal microscopy was used to determine the cellular distribution of TGFα and ADAM17 in NHBE cells following stimulation with IL-13 for various lengths of time. Representative images from cultures of NHBE cells treated with media only (control) or IL-13 (10 ng/ml) for 15 or 30 min are shown. NHBE cultures were imaged in Z-stack mode from the basal to the apical boundaries of the cells. Images shown are x-y planes (large squares) halfway between the basal-most and the apical-most images, bordered by corresponding y-z planes (shown at right of x-y plane) and x-z planes (shown at bottom of x-y plane). The y-z and x-z plane images are from the sites indicated by the white arrows at the bottom and the right of the x-y plane images, respectively. a → b denotes the apical (a) to basal (b) direction as it relates to the x-z and y-z planes. TGFα (red) and ADAM17 (green); scale bars represent 10 μm.

**Figure 6:**
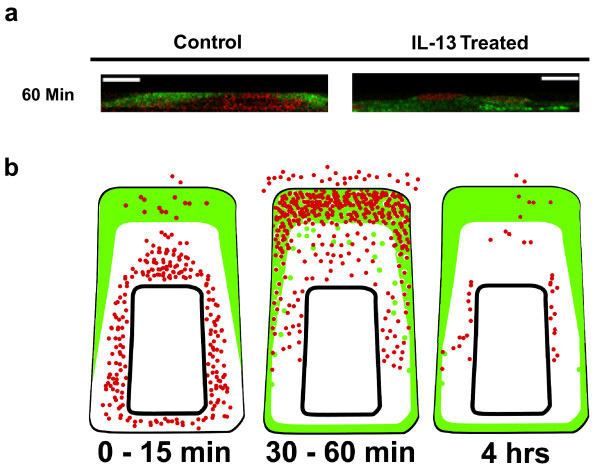
**Summary of TGFα and ADAM17 expression patterns induced by IL-13**. **a) **Confocal images (y-z plane; apical to basal cross-section) of NHBE cells exposed for 60 min to media alone (control) or IL-13 (10 ng/ml). See Additional files 1 and 2 for movies of Z-stack images (basal to apical) taken from a control culture and an IL-13-treated culture, respectively, at this time point. TGFα (red) and ADAM17 (green); scale bars represent 10 μm. **b) **Illustration summarizing expression patterns of TGFα and ADAM17 observed via confocal microscopy in IL-13-treated NHBE cells at the times indicated. Colors represent TGFα (red) and ADAM17 (green).

Untreated NHBE cells (data not shown), or NHBE cells exposed only to control media (Figs. 5 and 6a; Table 1), were found to express TGFα and ADAM17 constitutively. The majority of the growth factor (TGFα) was localized to the interior of the epithelial cells, with ample expression observed in the basal and central regions of the cells. Little expression of TGFα was observed in apical cellular regions. By contrast, ADAM17 was expressed throughout the cytoplasm, although the majority of this enzyme was expressed in portions of the cytoplasm adjacent to the cell membrane, with expression particularly prominent in the apical region of the epithelial cells. In fact, about 80% of control cells exhibited this pattern of expression which remained relatively unchanged as NHBE cells were exposed to fresh media for 15 min, 30 min, 1 hr, or 4 hrs (Figs. 5 and 6a; Table 1). More precisely, the percentage of media-exposed, control cells exhibiting this expression pattern (TGFα interior with ADAM17 highly expressed in the apical region) at these time points was 81%, 82%, 71%, and 88%, respectively (see Table 1). The cross-section and Z-stack video images of the control cells (Fig. 6a and Additional file 1, respectively) as well as an illustration of a control cell (Fig. 6b) summarize the observed location of TGFα (red) and ADAM17 (green) in cells without IL-13 stimulation.

While exposure of NHBE cells to IL-13 for 15 min did not alter the location of TGFα expression compared to its location within control cells (Fig. 5), continued exposure to IL-13 for 30 min or more did induce an alteration in the location of TGFα expression. Specifically, at 30 min, patches of TGFα were less defined within the cytoplasm, with almost no TGFα expression detectable in the basal areas of IL-13-exposed cells. Rather, the majority of the growth factor was expressed in the apical region and on the apical surface of the NHBE cells (Fig. 5). This pattern of apical TGFα localization was observed in 46% of the IL-13-treated cells compared to just 18% of the control cells (Table 1). While IL-13 induced increased apical localization of TGFα, apical localization of ADAM17 was observed in fewer cells (54% compared to 82% of control cells) following IL-13 exposure, with the enzyme now found to a greater extent in the middle and basal regions of the NHBE cells. Thus, it would appear that when NHBE cells are exposed to IL-13, localization of TGFα shifts to the apical region of these cells within 15 to 30 min. Such a finding would be consistent with the movement of TGFα from its intracellular region of constitutive expression (middle and basal) into the apical region of these cells, a region where prominent ADAM17 expression is observed constitutively.

Following exposure of NHBE cells to IL-13 for 60 min, the expression patterns of both TGFα and ADAM17 remained similar to those observed in cells exposed to IL-13 for 30 min (Fig. 6a; Table 1; see Additional file 2); more treated cells expressed TGFα in their apical regions (35% compared to 29% of control cells) while fewer treated cells expressed ADAM17 apically (65% compared to 71% of control cells). However, the percentage of affected cells appeared somewhat intermediate between the 15 min and the 30 min-treated values. This finding may suggest that the TGFα, whose apical expression was induced by IL-13, is beginning to be cleaved from the cell, while ADAM17 is being internalized.

Following a 4-hr exposure to IL-13, little TGFα remains within most of the NHBE cells. In fact, 98% of the treated cells, compared to 88% of the control cells, express mainly ADAM17 with little to no TGFα expression found at any level within the cells. The relatively small amount of growth factor that is present appears to be expressed in the intracellular regions where TGFα was maintained prior to stimulation (middle or basal region of the cells). Conversely, more control cells (12%) express TGFα in their apical regions compared to IL-13-treated cells (2%). This dramatic shift from 35 – 46% of IL-13-treated cells expressing TGFα apically at 30 – 60 min, to just 2% of the treated cells expressing it at 4 hrs, is consistent with the apical TGFα being cleaved and released from the cells.

Taken together, the confocal images (examples provided in Figs. 5 and 6a) and quantitative analysis (Table 1) of TGFα and ADAM17 expression in NHBE cells support the conclusion that IL-13 can induce movement of a stored growth factor (TGFα) from the central and basal cytoplasmic regions to the apical region of airway epithelial cells, where it is cleaved by ADAM17. Fig. 6b illustrates the timing of this inducible translocation, with an increase in TGFα near the apical surface observed by 30 – 60 min of IL-13 exposure, with the growth factor co-localizing with ADAM17 in this region. By 4 hrs of IL-13 exposure, very little TGFα is observed within the cells, likely due to its being cleaved from the apical surface by ADAM17, following its IL-13-induced translocation.

## Discussion

In this study, we report what appears to be the first cytokine-induced redistribution of a growth factor (TGFα) from an intracellular store to the apical surface of a cell, where a protease required for shedding of the growth factor (ADAM17) is prominently expressed. Having demonstrated previously that IL-13-induced proliferation of NHBE cells is mediated by TGFα [6], this report extends those results by establishing that ADAM17 is required for both IL-13-induced proliferation and TGFα shedding in these cells. This conclusion is supported by data demonstrating that the proliferation and growth factor shedding are inhibited by antisense oligonucleotides directed against ADAM17, while rhADAM17-induced proliferation of NHBE cells can also be blocked with neutralizing anti-TGFα antibodies. In examining the mechanism whereby IL-13 induces these ADAM17-mediated events, a dramatic activation of ADAM17 was not observed; rather, IL-13 induced a change in the location of TGFα expression in 30 to 60 min, with expression shifted to the apical region of the NHBE cells where significant ADAM17 expression is observed constitutively. A slight increase in the expression of ADAM17 was also observed within the middle and basal regions of the cells following IL-13 stimulation; this observed increase may be relative, as it is possible that apically-located sheddase is released along with the cleaved growth factor. Alternatively, ADAM17 may be internalized, an event known to occur with PMA-stimulation [46].

While a short exposure to IL-13 appears to induce a rapid redistribution of TGFα in NHBE cells, by 4 hrs of exposure to the cytokine only a small amount of the growth factor is observed within the cells and that within the basal region. While low-level synthesis of TGFα may occur continuously in NHBE cells, regardless of stimulation, it is also possible that new intracellular stores of the growth factor must be synthesized following IL-13-induced cleavage of apically-located TGFα.

Implications of the novel IL-13-induced mechanism directing TGFα to the apical region/surface of NHBE cells are broad-reaching, having the potential to provide insight not only into the role of epithelial cells in allergic asthma, but also into the impact of intracellular growth factor pools in a variety of cell types and diseases. Such intracellular stores are known to exist in neutrophils and monocytes where TGFα appears to be stored in membrane-bound compartments [33]. Intracellular stores of EGF have been similarly reported in human submandibular and parotid glands [47,48]. There is not, however, a complete understanding of the cellular mechanisms activating these stores, particularly in response to inflammatory stimuli.

By contrast, some growth factors, rather than being stored in intracellular compartments, are known to sort to various surfaces of polarized epithelial cells immediately following translation. For example, in Madin-Darby canine kidney cells, pro-TGFα sorts to the basolateral surface in a process requiring specific domains within the newly translated protein [49] and interaction with specific cytoplasmic proteins [50]. Similar sorting of another EGF family ligand, heregulin-α, also appears to occur in human bronchial epithelial cells [51]. EGF, however, has been found to sort to both apical and basolateral surfaces of polarized epithelial cells where it is released into the medium surrounding the cells. Differential activation of this growth factor then occurs due either to the presence, or activity, of metalloproteinases within the extracellular compartments around the cells [52].

In a similar fashion, the constitutive expression of activated ADAM17, occurring mainly in defined apical and lateral regions of NHBE cells, could result in constitutive release of TGFα during exponential and stationary growth of these cells. Constitutive release of TGFα is observed in unstimulated NHBE cells in vitro [6], where it appears to be mediated by ADAM17 (Fig. 3c). Although the present study does not distinguish the continuous presence of a small amount of TGFα in the cell membrane from a slow sorting of intracellular growth factor to this membrane, it does indicate that TGFα present in the membrane of a resting cell can be cleaved when it encounters activated ADAM17. Specifically, addition of a large excess of exogenous rhADAM17, which ensures a high probability of cleaving all TGFα in the membrane, results in a significant increase in soluble TGFα compared to control levels (Fig. 1a). This cleavage and release of TGFα by exogenous ADAM17 is similar to that observed previously using cell membrane preparations [38].

While constitutive release of TGFα may be important for general maintenance of an epithelial barrier, it is the inducible nature of TGFα redistribution that likely contributes to the role of airway epithelial cells as rapid "effectors" following a provocation, such as inhalation of an allergen to which the host is sensitized. By maintaining intracellular reserves of growth factors, and perhaps other molecules, as well as the constitutive expression of proteases that activate these factors, the reaction time in response to inflammatory stress and other epithelial injuries can be minimized. This inducible system also provides a number of safeguards to ensure the cell will be both equipped to respond to a stimulus and to direct that response in a specified manner. For example, the maintenance of intracellular growth factor reserves eliminates the possibility of surface-tethered molecules being inadvertently cleaved prior to their being needed for response to a specific biological insult. Such unintentional cleavage events could occur as neutrophil elastase or other proteases become present in the airway as a natural response due to infiltration of inflammatory cells following inhalation of everyday irritants. If growth factors were expressed constitutively in large amounts on airway epithelial cells, such proteases might liberate ligands such as TGFα, resulting in unwarranted consequences such as upregulation of mucin gene expression [53] or unnecessary proliferation.

The IL-13-inducible, apparent movement of TGFα from intracellular basal regions to the apical region/surface of NHBE cells could also have evolved as a way to lessen the impact of TGFα on cell types which underlie the epithelium. By keeping the ligand and sheddase separated physically within the epithelial cells, cleavage of the growth factor is prevented; even direct PKC activation, an event known to enhance ADAM17 activity and subsequent shedding [26,43], was incapable of inducing TGFα release above constitutive levels in this study. Inducible movement of the growth factor into the apical region where activated ADAM17 is present, however, would direct the shedding of TGFα exclusively from the apical surface of the NHBE cells toward neighboring epithelial cells, or resident and infiltrating inflammatory cells within the epithelium, rather than toward the basally-located fibroblasts or smooth muscle cells. In this manner, the IL-13-induced mechanism may provide a means of maximizing the presence of growth factor near damaged epithelial cells in an inflamed airway, enhancing the probability of epithelial barrier restoration without induction of remodeling features such as fibrosis or smooth muscle hyperplasia. A related mechanism has been suggested previously when heregulin-α was observed to be present exclusively in the apical membrane of human airway epithelia while its receptors, erbB2-4, were found to be present only on the basolateral membrane [51]. This arrangement appears to allow for ligand-receptor interaction only after epithelial integrity is disrupted, or when the tight junctions between cells are opened.

## Conclusion

In NHBE cells, IL-13-induced proliferation and TGFα shedding are mediated by ADAM17. Surprisingly, IL-13 does not seem to regulate these events by inducing a dramatic activation of ADAM17; rather, the cytokine appears to initiate a change in location of TGFα expression to the apical region of the cells where ADAM17 is prominently expressed. Thus, the cytokine appears to induce redistribution of an intracellular store of TGFα into a location where ADAM17 is expressed constitutively, thereby directing the apical cleavage and shedding of the growth factor.

Since growth factors exhibit their functions during many stages of development, cellular differentiation, the healing process, and inflammatory responses, the finding that stored growth factors can be released from cells in response to cytokines is likely to have far-reaching impact. Such cytokine-induced release may prove essential for restorative biological functions, yet also mediate deleterious cellular outcomes as growth factor levels are enhanced repeatedly during chronic inflammation. Thus, while the precise mechanism whereby IL-13 induces the movement of TGFα to the apical surface of NHBE cells remains to be elucidated, unraveling such a mechanism will likely provide diverse therapeutic targets for the prevention of airway remodeling or the enhancement of epithelial repair.

## Abbreviations

IL = interleukin

TGFα = transforming growth factor alpha

ADAM = a disintegrin and metalloproteinase

TNFα = tumor necrosis factor alpha

TACE = TNFα converting enzyme

NHBE = normal human bronchial epithelial

ALI = air/liquid interface

ELISA = enzyme-linked immunosorbent assay

PKC = protein kinase C

NO = nitric oxide

MAP kinase = mitogen activated protein kinase

Erk = extracellular signal regulated kinase

PMA = phorbol-12-myristate 13-acetate

rh = recombinant human

EGFR = epidermal growth factor receptor

TIMP = tissue inhibitor of metalloproteinase

## Competing interests

The author(s) declare that they have no competing interests.

## Authors' contributions

BWB performed the majority of the studies, participated in study design and data interpretation, and drafted the manuscript. TS prepared RNA samples as well as performed and interpreted the RT-PCRs. ELM helped design, and performed the quantitative analyses of the confocal studies. LDM provided input and oversight regarding all aspects of study design and interpretation of results. She also was responsible for revising and finalizing the manuscript. All authors read and approved the final manuscript.

## Supplementary Material

Additional file 1 Confocal Z-stack images of NHBE cells exposed to control media for 60 min. NHBE cells were exposed to control media for 60 min, reacted with fluorescent-tagged antibodies against TGFα (red) and ADAM17 (green), as described in Materials and methods, and then imaged by confocal microscopy. Shown is a video of the Z-stack images beginning with the basal-most section of the NHBE cells and ending with the apical-most section. Click here for file

Additional file 2 Confocal Z-stack images of NHBE cells exposed to IL-13 for 60 min. NHBE cells were exposed to IL-13 (10 ng/ml) for 60 min, reacted with fluorescent-tagged antibodies against TGFα (red) and ADAM17 (green), as described in Materials and methods, and then imaged by confocal microscopy. Shown is a video of the Z-stack images beginning with the basal-most section of the NHBE cells and ending with the apical-most section. Click here for file
